# Understanding the Stony Bridge between Osteoporosis and Vascular Calcification: Impact of the FGF23/Klotho axis

**DOI:** 10.1155/2021/7536614

**Published:** 2021-08-30

**Authors:** Xu Wei, Xinyi Huang, Ning Liu, Baoyu Qi, Shengjie Fang, Yili Zhang

**Affiliations:** ^1^Wangjing Hospital, China Academy of Chinese Medical Sciences, Beijing, China; ^2^School of Traditional Chinese Medicine, Beijing University of Chinese Medicine, Beijing, China; ^3^School of Traditional Chinese Medicine & School of Integrated Chinese and Western Medicine, Nanjing University of Chinese Medicine, Nanjing, China

## Abstract

A relationship between osteoporosis (OP) and vascular calcification (VC) is now proposed. There are common mechanisms underlying the regulation of them. Fibroblast growth factor- (FGF-) 23 and Klotho are hormones associated with the metabolic axis of osteovascular metabolism. Most recently, it was suggested that the FGF23-klotho axis is associated with increasing incidence of fractures and is potentially involved in the progression of the aortic-brachial stiffness ratio. Herein, we discussed the potential role of the FGF23/Klotho axis in the pathophysiology of OP and VC. We want to provide an update review in order to allow a better understanding of the potential role of the FGF23/Klotho axis in comorbidity of OP and VC. We believe that a better understanding of the relationship between both entities can help in proposing new therapeutic targets for reducing the increasing prevalence of OP and VC in the aging population.

## 1. Introduction

Osteoporosis (OP) is a systemic skeletal disease, characterized by low bone mass and deterioration in the microarchitecture of the bone tissue, which has greater susceptibility to fractures [1]. The prevalence of this metabolic bone disease is very high in the population, and fractures resulting from osteoporosis are becoming frequent situations. Vascular calcification (VC) in the past was reported a passive process, traditionally considered a degenerative consequence of aging [2]. However, recent studies have identified it is an active process, which has histopathological characteristics, mineral composition, and initiation and development mechanisms characteristic of bone formation [3]. Paradoxically, patients suffering from OP exhibit many of the classic hallmarks of vascular calcifications, which are known as “calcification paradox” [4].

There has been growing researchers in studying the relationship between metabolic bone disease and cardiovascular diseases over the years. Current clinical practice evidence supports the notion that patients with OP frequently suffer from VC, which was shown as a predictor for the progression of cardiovascular morbidity/mortality and osteoporotic fractures [5]. In the famous Framingham Heart Study, a 30-year longitudinal analysis of bone loss and VC showed that cortical bone loss measured at the metacarpal was related to the progression of atherosclerotic aortic calcification in women [6]. The association between cardiovascular disease and calcium deposition in the vasculature was first described in the 19th century [7]. Traditionally, OP and VC have been considered as independent processes related to age; although, a correlation has been demonstrated between the loss of bone mass and vascular calcification through a series of recent epidemiological studies [8–10].

Another important concept that connects OP and VC is the bone-vascular axis. Growing evidence linking bone with different functional and structural characteristics of arterial tree has contributed to developing the concept of the bone-vascular axis [11]. The emerging new study results still continue to enhance the rationality of the bone-vascular axis, which is reflected in the interpretation and discovery of the interaction mechanism [12–14]. What is more, clinical evidence demonstrated that some medications such as statins, insulin, and antihypertensives could exert protective effects on both osteoporosis and cardiovascular disease, suggesting a common pathophysiological basis [15].

Over the past decade, although there is a complex relationship between OP and VC, various research tools enabled clinicians and scientists to take a closer look at the molecular and cellular mechanisms behind these two diseases [16]. Among them, the knowledge progress of the FGF23/Klotho axis and its influence on phosphorus and calcium metabolism change our vision of the regulation of mineral metabolism in the past few years [17]. Fibroblast growth factor- (FGF-) 23 and Klotho are hormones associated with the metabolic axis of osteovascular metabolism. Klotho is known as a membrane protein in response to the effect of FGF-23 on kidney [18]. The FGF-23, which is a growth factor synthesized in the bone, acts in the renal to inhibit activation of vitamin D and to induce Pi excretion through proximal tubular epithelial cells [19]. Most recently, it was especially investigated that the FGF23-klotho axis is associated with increasing incidence of fractures and is potentially involved in the progression of aortic-brachial stiffness ratio [20]. Furthermore, an essential role of the FGF-23 is in the positively regulation and modulation of the OPG gene expression, emphasizing its role in the bone-vascular axis [21]. While in animal models, Klotho also has been reported to exert various effects on bone formation and to play a protective role in vascular calcification [22, 23].

Hence, this review provides an update in order to allow a better understanding of the potential role of the FGF23/Klotho axis in comorbidity of OP and VC. We believe that a better understanding of the relationship between both entities can help in proposing new therapeutic targets for reducing the increasing prevalence of OP and VC in the aging population.

## 2. Mechanism of the FGF23/Klotho axis on Osteoporosis

### 2.1. Vitamin D

FGF23 was later demonstrated to be involved in the decreases of 1,25(OH)_2_ D in both global and tissue-specific deletion studies [24]. The renal 1*α*(OH) ase enzyme is recognized as the major source of circulating 1, 25(OH)_2_ D, which is tightly downregulated by the phosphaturic hormone FGF23. It is well established that activation of the FGF23/*α*-klotho signal is able to inhibit the production of 1,25(OH) _2_ D by suppression of the renal Cyp27b1 expression. In addition, diverse studies point to these active forms, including 1,25(OH) 2 D and 25(OH) D, can be metabolized largely by CYP24A1, while FGF23 could increase the expression of CYP24A1 [25]. It is well known that 1,25(OH)2 D, a steroidal hormone, acts mainly as a ligand of the widely distributed nuclear vitamin D receptor (VDR). Vitamin D compounds reduce the RANKL/OPG ratio and osteoclast differentiation by acting on VDR which is preferentially expressed in osteoblasts and osteocytes in the bone, while mineral-regulating hormones such as PTH and FGF23 plays a complex role in the VDR-regulated expression of RANKL and OPG in osteoblast lineage cells [26]. Also, interesting results were observed in a recent animal experiment. Klotho −/− VDR *Δ*/*Δ* knockout mice, like Klotho −/− -PTH −/− mice, had complete rescue of the skeletal phenotype, while FGF23 −/− VDR *Δ*/*Δ* knockout mice did not [27]. These data provided a second line of evidence that Klotho and FGF23 effects independently in bone.

### 2.2. Phosphate (Pi)

FGF23 reduces serum Pi by inhibiting 1,25-dihydroxyvitamin D synthesis, suppressing intestinal Pi absorption, and by downregulating the transporters NPT2a and NPT2c, suppressing Pi reabsorption in the proximal tubules [28]. Of special interest, the organ-specific expression of Klotho has been detected that is practically confined to distal tubules, which is also the site for initial FGF23 binding and signaling. The mechanism of how signals originating in the distal tubules translate into the proximal tubules to reduce Pi reabsorption remains unknown. However, recently, a novel mechanism of efflux of inorganic Pi from renal proximal tubular epithelia has been proposed: Xenotropic and polytropic retrovirus receptor 1 mediates efflux of Pi through the basolateral membrane of the renal proximal tubular epithelia [29]. Furthermore, an in vivo experiment implied a potential role for the FGF23/Klotho/PTH axis in the Pi handling in the proximal tubules [30].

It is confirmed by several studies that a high phosphorus diet decreases the *α*-Klotho expression. On the contrary, the expression of *α*-Klotho increased by a Pi-deficient diet in mature mice [31]. One recent study further explored dietary Pi exerts effects on the expression of *α*-klotho at different life stages, which allowed to hypothesize that there are more adverse effects in weaned mice given high Pi diet on the renal *α*-klotho expression and pathogenesis of renal calcification compared with periadolescent mice given the same diet [32]. Meanwhile, the main regulatory mechanism of enhanced FGF23 production by high dietary Pi is the posttranslational modification of FGF23 protein through a gene product of GALNT3. Furthermore, high extracellular Pi directly activates FGFR1, and its downstream intracellular signaling pathway regulates the expression level of GALNT3 [33]. The capability of increasing FGF23 production in response to high Pi has highlighted the important role of the FGFR1c-GALNT3 axis [34].

### 2.3. Parathyroid Hormone (PTH)

FGF23, which is a hormone synthesized mainly in mature osteoblasts and osteocytes, exerts inhibitive effects on the production of 1,25(OH) 2 D and PTH [35]. Recent clinical studies suggest a very intriguing opposite independent association between PTH and FGF23: premenopausal women exhibited a positive relationship, while FGF23 has a negative relation with PTH in men. To date, the nature of the regulation involved in the links between FGF23 and PTH is not clearly defined, as PTH administration increases circulating FGF23 in some studies, while decreasing it in others [36, 37]. A reasonable explanation is the dual antagonistic effect of FGF23 on parathyroid cells; that is to say, FGF23 directly inhibits the secretion of PTH and simultaneously inhibits the renal vitamin D activation, thus indirectly stimulating the synthesis of PTH [38]. In addition, the FGF23/Klotho axis has been proposed that it is not essential for the phosphaturic and anabolic functions of PTH by using the novel FGF23 and Klotho knockout mice in other experiments, which further represented PTH as one of the most promising new therapeutic targets for improving the skeletal quality of patients even in the presence of abnormal serum FGF23 levels [39].

### 2.4. Ectonucleotide Phosphatase/Phosphodiesterase 1 (ENPP1)

ENPP1 was the first enzyme identified and is the only human enzyme capable of generating extracellular pyroPi [40]. Recently, clinical study reported a cohort of middle age men with hereditary early onset osteoporosis (EOOP) manifested by vertebral and/or radial fractures and low bone mass with heterozygous ENPP1 deficiency. Specifically, these patients exhibit mildly elevated FGF23, decreased serum phosphorous, elevated urinary Pi clearance, and radiographic evidence of osteoporosis [41]. This allowed to hypothesize the suppression of murine FGF23 and Alp by human ENPP1-Fc, which was demonstrated later by animal experiments [42]. Other study also showed that Enpp1 loss significantly downregulates the renal Klotho expression under the elevated levels of dietary Pi. Meanwhile, a progressive increase in serum Pi levels promote FGF23 production from the bone, but the FGF23 signals are altered due to the decreased Klotho expression [43].

### 2.5. Bone Marrow-Derived Mesenchymal Stem Cells (BMSCs)

BMSCs are primarily secreted from mesoderm with infinite expansion and differentiation into the nerve, muscle, and bone. It plays a significant role in bone formation and repair. One latest original study clarified that astragalus exerts influence on the aging BMSC model and on the vitamin D-FGF23-Klotho axis. In fact, it has been verified that changes in astragalus concentration affect diverse factors such as FGF23, Klotho, and CYP24A1. After adding serum-containing astragalus, the activity of cells and the osteogenic ability was increased; the expression levels of FGF23, Klotho, and CYP24A1 were decreased, while the expression levels of CYP27B1 were increased. The trend was more obvious with a progressive increase in astragalus concentration, which finally demonstrated that astragalus has the ability of inhibiting the aging of BMSCs and improving the osteogenesis ability by regulating the VD-FGF23-Klotho pathway. In addition, several evidences point to secreted Klotho, through the inhibition of FGFR1 and ERK phosphorylation, and can delay the differentiation of human mesenchymal stem cells into osteoblasts [44].

### 2.6. Wnt Signaling

Historically, the participation of the Wnt/*β*-catenin pathway in bone disorders has been widely documented. WNT1 in the bone is secreted from osteocytes, and WNT1 mutations may be direct players in the process of impaired WNT/*β*-catenin signaling and decreased bone formation [45, 46]. However, the mechanisms involved in the direct interaction of FGF23 or Klotho with Wnt elements are not clearly defined. It has been shown that the extracellular domain of Klotho binds to multiple Wnt ligands, specifically inhibiting their ability to activate Wnt signaling [47, 48]. Carrillo et al. identified that FGF23 plays a directly role of inhibiting Wnt signaling through the increase of Dkk1 levels with the participation of soluble Klotho in the bone [49]. Recently, some studies have shown that there is a positive correlation between FGF23 and sclerostin levels in patients with rheumatic arthritis; in this sense, these indicated a direct relationship among FGF23, reduced Wnt activity, and bone demineralization in these patients [50]. In vitro studies, the link between FGF23, Klotho, and Wnt signaling in bone cells had been recently confirmed [51]. Klotho is located within osteocytes and osteoblasts, suggesting that the bone is another target organ for FGF23. Several studies indicate that Klotho as a negative modulator of bone formation [23]. Moreover, the capability of FGF23 with the assistance of klotho is to inhibit Wnt signaling and osteogenesis by enhancing the Dkk1 expression, which is supported by previous observations showing that Wnt activity is increased in Klotho knockout mice [52]. Additionally, Ma et al. observed that in UMR-106, a bone cell line, the addition of *β*-glycerophosphate upregulates the expression of Wnt target genes; the coadministration of *β* -glycerophosphate and sKlotho results in a decrease in FGF23 levels and a reduction in Wnt activation, suggesting that sKlotho may modulate osteogenesis and FGF23 production [53].

### 2.7. NF-*κ*B Signaling Pathway

In fact, it has been verified that the NF-*κ*B overexpression can promote apoptosis of vascular smooth muscle cells and inhibit cell proliferation [54]. Additionally, NF-*κ*B inhibition results in osteoblast differentiation and bone enhancement [55]. Furthermore, it has been described that activation of NF-*κ*B by palmitate can induce apoptosis of MC3T3-E1 osteoblasts [56]. Growing recent evidence suggests that klotho has inhibitory effects results in the degradation of I*κ*B and activation of nuclear NF-*κ*B [57]. In latest article, the authors reported that the NF-*κ*B inhibitor pyrrolidine dithiocarbamate (PDTC) participates in reducing the number of apoptotic cells and attenuating the activity of caspase-3 induced by DEX, suggesting the involvement of NF-*κ*B in DEX-induced MC3T3-E1 apoptosis [58]. Klotho was demonstrated to be involved in the inhibition of NF-*κ*B activation and the reduction of the DEX-induced caspase-3 expression by these researchers, in consistent with previous studies. These results indicate that NF-*κ*B activation may mediate the antiapoptotic effect of Klotho and proapoptotic effect of DEX.

### 2.8. Mechanism of the FGF23/Klotho axis on Vascular Calcification

Patients with advanced CKD were frequently found to have VCs, and at present, it is responsible for the high CVD-related mortality [59]. VC is the final consequence of a process where VSMC is transdifferentiated into osteoblast-like cells [60]. The molecular mechanisms of VC present substantial similarities with those of skeletal bone mineralization.

### 2.9. Phosphate

The involvement of elevated FGF23 in the progress of VC is still under debate [61, 62]. A correlation has been demonstrated between higher FGF23 levels and increased aortic calcification in clinical studies of 65 hemodialysis and 142 patients with CKD stages 2–5 [63]. In contrast, different results were observed in a much larger cohort study named the CRIC (Chronic Renal Insufficiency Cohort), which performed in a cohort of 1501 patients with a mean eGFR of 47 mL/min/1.73 m^2^ and showed that there are no associations between FGF23 levels and the prevalence of coronary artery calcification [64]. The CRIC study was further supported by in vitro experiments. Stimulation with FGF23 did not augment phosphate-induced calcification, neither in human vascular smooth muscles cells (VSMCs) in dependence on the Pi concentration nor in aortic rings in the presence of soluble Klotho [64]. Other experimental studies even suggested that FGF23 exerts protective effects on the progression of VC [65]. Recently, Chen et al. further showed that the overexpression of FGF23 and Klotho in rat VSMCs attenuated phosphate-induced calcification by inhibiting Wnt7b/*β*-catenin signaling [66]. On the contrary, Jimbo et al. reported FGF23 may dedicate an enhanced phosphate-induced calcification in Klotho-overexpressing VSMCs [61]. In other studies, a remarkable aspect is that FGF23 is recognized as a protective factor, since it was observed that depletion of FGF23 levels in rodent CKD models results in a more severe VC [67].

### 2.10. Intermedin 1–53

Intermedin (IMD) is characterized by a potential endogenous protective peptide of the cardiovascular system, activating the cyclic adenosine monophosphate (cAMP)/protein kinase A (PKA) pathway [68]. Previous studies showed that the levels of IMD downregulated in calcified aortas in vivo and in calcified VSMCs, and IMD_1–53_ treatment was later demonstrated to be involved in the alleviation of vascular calcification [69]. However, a novel mechanism behind the suppression of IMD_1–53_ on VC [70] has been proposed by Chang et al. In their study, IMD_1–53_ administration has been shown to attenuate VC, suppress osteoblast-like cell formation, and increase the expression of Klotho in the aorta of CKD rats. To directly examine the effect of IMD_1–53_ on Klotho and calcification in VSMCs, IMD_1–53_ was applied to cultured VSMCs. An interesting and more importantly aspect is that IMD_1–53_ increased the level of vitro Klotho protein in calcified VSMCs. Klotho knockdown blocked the inhibitory effect of IMD_1–53_ on calcification in VMSCs and transformation of VMSCs into osteoblast-like cells [71]. Taken together, in the basis of the vivo and vitro findings, we consider the numerous processes in which IMD_1–53_ is involved, including as stimulator to upregulate renal, vascular, and plasma Klotho protein levels.

### 2.11. Peroxisome Proliferator-Activated Receptor *γ* (PPAR *γ*)

It has been indicated that mice lack Klotho promoted calcification and osteoblastic differentiation of VSMCs [72], whereas Klotho transgenic mice were observed a significant decrease in the incidence of calcifications and have better preserved renal function compared to wild-type mice with CKD [73]. It has been suggested that Klotho suppresses osteoblastic transdifferentiation and calcification of VSMCs by inhibiting PiT-1/2-dependent Pi uptake, thus repressing the expression of Cbfa1/Runx2. Klotho has been identified as a target for nuclear receptor peroxisome proliferator-activated receptor (PPAR) *γ* [74]. Thiazolidinediones, which act as PPAR *γ* agonists, participate in the upregulated Klotho expression in HEK293 cells and several renal epithelial cell lines at the mRNA and protein level. This induction was blocked by siRNA-mediated gene silencing of PPAR *γ* or PPAR *γ* antagonists, which have been shown to attenuate high glucose-induced VSMC calcification [75]. The latest findings suggested that Klotho plays a modulatory role in the regulation of Pi-induced vascular calcification by PPAR *γ*, since it has recently been shown that knockdown of Klotho abolished the ability of activated PPAR *γ* to inhibit calcification in VSMCs. These findings suggested a potential mechanism of PPAR *γ* in the regulation of Pi-induced vascular calcification [76].

### 2.12. Salusin-*β*

Salusins are characterized as two related peptides: salusin-*α* and salusin-*β* [77]. In contrast to salusin-*α*, associations traditionally considered are salusin-*β* referred to the regulation of cardiovascular and the accelerated development of atherosclerosis [78]. Salusin-*β* was expressed at high levels in macrophage foam cells, VSMCs, and fibroblasts within atherosclerotic lesions in coronary arteries [79]. CD36 lymphocytes have also been confirmed to play a key role in the formation of foam cells and participate in the uptake of oxidized low-density lipoprotein by macrophages. The interaction between these two key molecules is a hot research topic at present [80]. Moreover, existing evidence demonstrated that salusin-*β* increased the expressions and activity of acyl coenzyme A-cholesterol acyltransferase-1 (ACAT-1), and silencing of ACAT-1 abolished the salusin-*β*-induced lipid accumulation. Salusin-*β* also stimulates the proliferation of VSMCs and fibroblasts and induces the expression of growth-associated genes, such as c-myc and c-fos [77]. These phenomena are regarded as important characteristics of atherosclerosis.

In addition, in light of oxidative stress, salusin-*β* is widely accepted as an oxidation inducer in cardiac tissues, VSMCs, and endothelial cells in multiple disease scenarios [81]. Klotho is characterized for antiaging that protects cells from inflammation and oxidative stress [82]. Klotho treatment exerts protective effects on the heart from hyperglycemia-induced injury by inactivating the reactive oxygen species (ROS) signaling pathway [83]. In particular, oxidative stress is a significant regulator in the expression of the Klotho gene, and an essential role of the associations of oxidative stress with klotho is in the process of VC [84]. However, the direct evidence involved in the links between salusin-*β* and Klotho is not clearly defined, and they might target the same oxidative stress signaling pathway. As demonstrated by recent study, Salusin-*β* regulates VC through activation of NAD(P)H/ROS-mediated Klotho downregulation, suggesting that salusin-*β* may represent one of promising new therapeutic targets for the treatment of VC [85].

### 2.13. Wnt7b/*β*-Catenin Pathway

The Wnt/*β*-catenin pathway is accepted as a family of proteins that is implicated in the regulation of many vital functions such as vascular calcification [86]. More recently, Yao et al. in a study revealed that high phosphorus level leads to aortic calcification via *β*-catenin in CKD [87]. Besides, VC in CKD was also reported to be induced via a mechanism involving the Wnt/*β*-catenin pathway. However, additional studies are needed to specifically address the mechanisms by which the Klotho/FGF23 axis could influence the relevant signaling molecules in Wnt/*β*-catenin signaling. Recent study demonstrated that both Klotho and FGF23 have opposite effects on the VSMC calcification induced by high phosphate. In addition, Klotho and FGF23 impaired phosphate-induced vascular calcification by inhibiting the Wnt7b/*β*-catenin pathway [66]. Other studies have shown that inhibition of the Wnt/*β*-catenin pathway is able to prevent VSMC calcification by Klotho supplementation [88].

**Figure 1:**
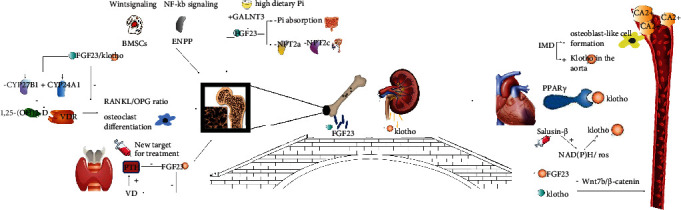
The common mechanism by which the FGF23/Klotho axis affects OP and VC.

The common mechanism by which the FGF23/Klotho axis affects OP and VC was shown in Figure 1.

## 3. Discussion

The link between OP and VC had been recently confirmed. Patients with OP frequently suffer from VC. Several studies have demonstrated that VC presents substantial similarities with OP, for example, they share several cardiovascular risk factors such as age, hypertension, dyslipidemia, diabetes, and cigarette smoking [5], the majority of studies of our review suggested that this relationship is not only due to the presence of common clinical risk factors but most probably also to underlying biological factors that connect them. Furthermore, a recent retrospective observational study promoted that it seems unlikely that VC could be secondary only to the catabolic processes in the osteoporotic bone, considering the lack of any significant impact of bisphosphonate therapy in the progression of VC [89, 90]. The existence of underlying biohumoral mechanisms indicates that the connection of the two pathological conditions is probable. In support of this, it has been proposed that vitamin D plays a modulatory role in the regulation of both bone metabolism and valve calcification [91]. Vitamin D is the precursor to the active steroid hormone of 1,25-dihydroxyvitamin D (1,25[OH]_2_ D), known as calcitriol, a key regulator of calcium and phosphorous homeostasis through actions in the intestine, kidneys, and bones. Activated vitamin D binding to the vitamin D receptor regulates transcription of hundreds of genes, including those involved in cell cycling, proliferation, differentiation, and apoptosis [92]. But interestingly, the results of studies [93, 94] exploring the association between bone mineral density and vascular calcification are inconsistent in terms of race. A study in different races revealed that vitamin D and/or vitamin D precursor levels in European Americans was correlated with BMD and negatively associated in African American [95]. In addition, positive correlations in African Americans between calcified atherosclerotic plaque and 25(OH)D levels have been reported; instead, the associations in European Americans were negatively [96]. Some researchers suppose that there are differences in terms of the appropriate “normal” range for 25(OH)D levels among racial and ethnic groups [97].

Nowadays, the FGF23/Klotho system is considered as a principal regulator of phosphate homeostasis, exerting its effects independently of the two classic endocrine regulating factors: PTH and calcitriol. PTH and calcitriol synthesis is also actively regulated by this system. Overall, the important role of the endocrine bone–kidney–parathyroid gland axis was highlighted by FGF23 to control serum phosphate levels by negative feedbacks over kidney and parathyroid glands [98]. In addition, the relationship between the FGF23/Klotho axis and Wnt signaling pathway has also been revealed [99]. Herein, we discussed the potential role of the FGF23/Klotho axis in the pathophysiology of OP and VC, which we believe that has great clinical value in the context of an aging society.

Although the preliminary basic research on VC hvs shown evidence of pathological mechanisms similar to OP, how will these findings from experimental bench benefit patients with OP and VC? Clearly, animal models have some immanent peculiarities, including differences in bone growth pattern and a relative resistance against atherosclerosis and arterial calcification; thus, further studies are needed to evaluate whether these results can be extrapolated from animal to human. However, progress in imaging techniques will allow higher resolution, shorter acquisition time, and more reasonable radiation exposure in noninvasive preclinical evaluation [100]. With the improvement of techniques, these are becoming more accessible and affordable and should be employed in clinical studies to assess simultaneously OP and VC. Additionally, intervention studies addressing the question whether treatment of OP benefits VC or vice versa would be desirable, based on the molecular and cellular concepts of VC.

To sum up, studies have revealed common pathogenesis underlying these two frequent age-related disorders by the combined skeletal and vascular phenotypes of animal models. However, additional studies are needed to demonstrate whether these findings can be directly extrapolated to the human OP/VC syndrome. Thus, these data highlight the need of clinicians to employ an open-minded approach with integrative thinking, with the aim to benefit patients with OP and VC.

## Data Availability

The data used to support the findings of this study are included within the article.
